# A model for a drug distribution system in remote Australia as a social determinant of health using event structure analysis

**DOI:** 10.1186/s12913-017-2629-x

**Published:** 2017-09-25

**Authors:** John P. Rovers, Michelle D. Mages

**Affiliations:** 1 College of Pharmacy and Health Sciences, Drake University, Des Moines, IA 50311 USA; 2 Department of Pharmaceutical and Administrative Sciences, Drake University, 2507 University Avenue, Des Moines, IA 50311 USA

**Keywords:** Drug distribution, Social determinants of health, Medication access, Australia, Rural and remote health, Pharmacy services, Quality improvement, Policy, Health professions education

## Abstract

**Background:**

The social determinants of health include the health systems under which people live and utilize health services. One social determinant, for which pharmacists are responsible, is designing drug distribution systems that ensure patients have safe and convenient access to medications. This is critical for settings with poor access to health care. Rural and remote Australia is one example of a setting where the pharmacy profession, schools of pharmacy, and regulatory agencies require pharmacists to assure medication access. Studies of drug distribution systems in such settings are uncommon. This study describes a model for a drug distribution system in an Aboriginal Health Service in remote Australia. The results may be useful for policy setting, pharmacy system design, health professions education, benchmarking, or quality assurance efforts for health system managers in similarly remote locations. The results also suggest that pharmacists can promote access to medications as a social determinant of health.

The primary objective of this study was to propose a model for a drug procurement, storage, and distribution system in a remote region of Australia. The secondary objective was to learn the opinions and experiences of healthcare workers under the model.

**Methods:**

Qualitative research methods were used. Semi-structured interviews were performed with a convenience sample of 11 individuals employed by an Aboriginal health service. Transcripts were analyzed using Event Structure Analysis (ESA) to develop the model. Transcripts were also analyzed to determine the opinions and experiences of health care workers.

**Results:**

The model was comprised of 24 unique steps with seven distinct components: choosing a supplier; creating a list of preferred medications; budgeting and ordering; supply and shipping; receipt and storage in the clinic; prescribing process; dispensing and patient counseling. Interviewees described opportunities for quality improvement in choosing suppliers, legal issues and staffing, cold chain integrity, medication shortages and wastage, and adherence to policies.

**Conclusion:**

The model illustrates how pharmacists address medication access as a social determinant of health, and may be helpful for policy setting, system design, benchmarking, and quality assurance by health system designers. ESA is an effective and novel method of developing such models.

## Background

The World Health Organization (WHO) defines the social determinants of health as, “… the conditions in which people are born, grow, live and age and the wider set of forces and systems shaping daily life. These systems include economic policies and systems, development agendas, social norms, social policies and political systems.” [1]. Healthy People 2020 notes that access to health services is an example of a social determinant of health [2]. WHO makes it clear that it is important to address social determinants of health that create barriers to good care. This would include health systems, of which medication distribution is a fundamental component [3].

Pharmacists are the health care professionals responsible for ensuring that people have access to the medications they need. To do so requires pharmacists to create systems for medication procurement, storage and distribution. This is consistent with the pharmacist’s role in addressing one of the social determinants of health, namely adequate access to medications. This responsibility becomes critical when pharmacists serve a poor, rural or remote population.

Australia is a wealthy country with a substantial indigenous population living in rural and remote areas, in poor socio-economic circumstances, and for whom access to medication may be limited [4]. Official data sources indicate that over 60% of the rural and remote Australian population is Aboriginal or Torres Strait Islander people [5]. More than 20% of indigenous Australians live in such locations. Forty-three percent of Aboriginal and Torres Strait Islander people are in the lowest quintile for gross household income. In rural and remote areas, 45–59% of residents in the lowest income quintile are indigenous. Spending on medications for Aboriginal and Torres Strait Islander people is approximately 44% of what is spent on non-indigenous Australians ($369 AUD versus $832 AUD). Access to pharmacists’ services is often inadequate, since there are 97 pharmacists per 100,000 population in major cities, compared to 60 per 100,000 in remote areas. Avoidable deaths are estimated as three times higher in Aboriginal and Torres Strait Islander people than in non-indigenous Australians [5].

Given this socio-economic situation, Australian schools of pharmacy, the profession itself, and relevant regulatory agencies acknowledge pharmacists’ responsibility for medication supply, especially to rural and remote Australia and to indigenous Australians.

The Australian Pharmacy Council is the accrediting body for pharmacy schools in Australia. The learning domains expected for an accredited degree program specify that all pharmacy graduates should have knowledge of, understanding of, and skills in, the sale and supply of medications, drug packaging and labeling, supply chains, medication stability, and rural and remote health systems, including Aboriginal Health Services [6].

The Pharmaceutical Society of Australia (PSA) is a professional body focused on improving pharmacy practice. Their code of ethics requires that a pharmacist:

“Supports the rights of all patients, including Aboriginal and Torres Strait Islander peoples, to access culturally safe and responsive, high quality professional services.”

and

“Promotes professional and environmental responsibility and accountability for the control, procurement, preparation, handling, supply, storage and disposal of therapeutic goods.” [7].

PSA has also developed a national competency standard framework for pharmacists [8]. It states that a competent pharmacist will maintain a logical, safe, and systematic dispensing system that includes procedures to maintain product stability, ensure proper labeling, and avoid errors.

Given the pharmacist’s responsibility for ensuring access to medications, especially in remote areas with a low socio-economic status, it is perhaps surprising that comparatively little research has been done on the drug procurement, storage, and supply systems in such settings. Research into the drug supply process would be useful for system design, policy making, health professions education, dispensing guidelines, benchmarking, and quality assurance. Yet, there are only a few studies that look at the process by which pharmacists supply medications. Early studies are limited to empirical descriptions of pharmacy systems, while more recent research was performed in a health-system setting in middle-income countries. Studies of systems in ambulatory care in remote and economically deprived areas of high-income countries appear to be sparse.

Two early papers described the role of pharmacists in the Indian Health Service, and isolated communities in Northern Canada [9, 10]. Winship describes some epidemiological, cultural, and practice aspects of working with the Navajo of Arizona [9]. Briggs discusses distribution of drugs by nurses, formulary development, stock control, and patient compliance in the Canadian Arctic [10]. Neither paper presents a formal model of a drug distribution system.

More recently, Kjos and co-authors developed a model for drug procurement, storage and distribution in four public hospitals in Vietnam [11]. Using WHO and International Pharmacists Federation (FIP) standards as a theoretical framework, they found that the system could be described using structural components created by the Ministry of Health, and functional components created by each hospital that permitted systems to be flexible enough to meet local needs.

Additional research in this area includes three studies in 29 Saudi Arabian Hospitals that examined prescribing, order transcription, dispensing, drug administration, drug monitoring, and patient education [12–14]. Most hospitals were found to have a drug formulary and a unit-dose drug distribution system.

A study of dispensing in four Brazilian hospitals found a number of problematic areas [15]. The work environment was felt to increase the risk for dispensing errors, while drug ordering, dispensing, and record keeping were also found to be sub-optimal.

In addition to these peer-reviewed evaluations of drug distribution systems, the grey literature includes a number of models and guidelines for drug distribution in poor, remote, or underserved areas.

A standard resource on managing access to medicines is available from Management Sciences for Health [16]. This resource is quite comprehensive in discussing numerous aspects of medication access including pharmaceutical manufacturing, drug importation policies, and pharmaceutical benefits in insurance programs. However, its appropriate applications for use are for poor countries in the Global South, not developed countries with existing medication distribution systems.

**Fig. 1:**
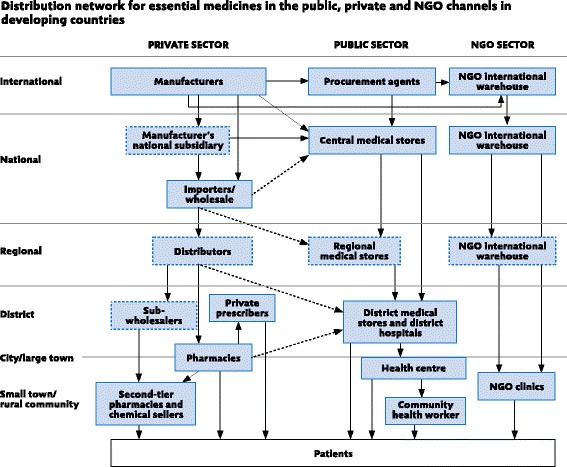
Distribution network for essential medicines in the public, private and NGO channels in developing countries. (Reproduced from Yadav with permission http://www.who.int/about/copyright/en

Yadav published a general model for the distribution of essential medicines in developing countries as shown in Fig. 1 [17]. Although the model provides an overview for how drugs may move through distribution channels in the private, government and non-governmental organization (NGO) sectors, it is insufficiently detailed to describe, design or evaluate any specific practice system.

WHO has created guidelines for drug procurement in developing countries that provide four strategic objectives and 12 operational principles for purchasing medications [18]. FIP’s Guidelines for Good Pharmacy Practice include advice on how to prepare, obtain, store, secure, distribute, administer, dispense, and dispose of medications [19]. Additional guidelines for the drug supply process in developing countries are also available for handling donated medications, drug storage in hot and humid environments, and training local workers on how to run a dispensing system [20–23].

One gap in the existing literature appears to be a description of how to design, manage, or evaluate the drug supply system for ambulatory patients in rural and remote regions of a high-income country where patients may live in very different cultural, social, and economic circumstances from their fellow citizens living in different urban or wealthier regions.

This study describes the drug supply system in such a setting using a novel, qualitative method known as Event Structure Analysis (ESA) [24, 25]. ESA has been suggested as a useful qualitative method for researchers who wish to identify and categorize data elements, and explore the connections between them [26]. Using ESA allows researchers to describe a model, while the rich data collected using qualitative methods allows them to examine the lived experiences of the health care workers who work in the model.

Although ESA does not appear to have been used in the pharmacy or other health services research literature, it has been used to study social change processes, in ethnographic studies, in historical sociology, in studies of organizational change, and in studies of cultural structures [27–31]. According to Abbot, ESA helps qualitative researchers create a formally coded narrative structure of the events in the data [25]. It allows a researcher to reduce the data in an extensive narrative to an inventory of actions and events that can be formalized in a manner that shows how they proceed from one another. ESA has been described as a form of “narrative positivism” that requires an analyst to apply formal rules to analyze narrative events [27]. The logical principles underlying ESA force analysts to be explicit in their reasoning which may result in studies that are more generalizable and reproducible than other qualitative methods [27]. For this study, ESA was chosen as the method of analysis in the hope that its requirement for analysts to use explicit reasoning and formal rules would be a particularly rigorous way of developing a model. By placing events into a model, the model becomes a visual representation of the drug procurement, storage and distribution system.

## Objectives

The primary objective of this study was to describe a model for a drug distribution and supply system in a rural and remote region of Australia. Drug distribution and supply was defined as the system of drug procurement, storage, and distribution [11]. The secondary objective was to learn the opinions and experiences of the healthcare workers who work in the system described.

## Methods

### Setting

The study setting was an Aboriginal community health service that cared for 2000 Aboriginal people living across 73,000 mile^2^ in 11 remote communities in The Central Desert [32]. The nearest communities of any size were Alice Springs (population 28,000) 600 miles to the east, and Kalgoorlie (population 32,000) 600 miles to the West. Although all patients were located a ten-hour drive to the west over the border with Western Australia, the health service administrative offices were located in the neighboring Northern Territory.

Each of the 11 remote communities had a clinic staffed by a remote area nurse (RAN). Four physicians and the Royal Flying Doctor Service (RFDS) provided medical support on-site (on a part-time basis) or remotely by telephone. RFDS also evacuated critically ill patients by air to the hospital in Alice Springs as needed. At the time of the study, the health service employed a full-time remote area pharmacist (RAP) who provided decentralized services to promote Quality Use of Medicines (QUM) [33]. QUM is an Australian national policy to promote access to the best possible drug treatment that is both effective and safe [33]. Since access to medicines is a fundamental component of QUM, as well as a social determinant of health, evaluating the drug product supply system is a question of interest. The question could be informally characterized as, “How do you get medication to people living 600 miles from the nearest pharmacy?”

### Data collection

Semi-structured interviews were carried out in English (by author JR) with a convenience sample of 11 individuals employed by the health service. Job titles and work locations of interviewees are included in Table 1. A community pharmacy in a regional capital city that served as the primary source of drug supply declined to be interviewed, but provided brief written answers to the interview questions. The interview questions are included in Table 2. The specific questions asked varied according to the responsibilities of each interviewee. Questions were developed using the FIP and WHO guidelines cited above as their theoretical basis, while follow-up questions were asked extemporaneously. Data saturation was achieved after only a few interviews. The study was approved by the Drake University Institutional Review Board, and all subjects gave written, informed consent for participation and publication of results prior to being interviewed.

**Table 1:** Interview Participants

Job title	Location
Health service continuity of care pharmacist^a^	Aboriginal Health Service Head Office
Health service chief executive officer	Aboriginal Health Service Head Office
Remote area nursing assistant^a^	Rural Clinic #1
Remote area nurse^a^	Rural Clinic #1
Remote area nurse^a^	Rural Clinic #2
Immunization and child health coordinator	Rural Clinic #2
Remote area pharmacist^a^	Work was decentralized, but based at Rural Clinic #2
Aged care coordinator	Rural Aged Care Facility
Director of pharmacy	Regional Capital City Hospital
Pharmacy technician	Regional Capital City Hospital
Health service clinical services assistant	Aboriginal Health Service Head Office

^a^Transcript used in ESA to create model

**Table 2:** Semi-Structured Interview Questions

Procurement	
1. Please tell me about how you decide which drugs to purchase.	
2. Please tell me about your drug tendering process.	
3. How do you make decisions about the quality of medicines you need to purchase?	
4. Please tell me about your audit processes.	
5. How do you decide on purchase quantities?	
6. Please tell me about your accounting processes that you use to pay your suppliers.	
7. How do you handle donated medicines?	
Storage	
1. Please tell me about your cold chain.	
2. Please tell me about how you keep medicines at the correct humidity.	
3. How do you monitor for expired products?	
4. How do you distribute drugs from the warehouse to the clinics?	
5. How do you handle returns of unwanted/damaged products?	
6. What process do you use to rotate stock?	
Distribution	
1. How do you order your medicines from central stores?	
2. How do you store your stock?	
3. How do you rotate your stock?	
4. How do you handle donated medicines?	
5. Tell me about how you dispense medicines to patients.	
6. What information do you give patients when you give them their medicines?	
7. How do you reconcile what you ordered from what you received from the warehouse?	
8. What cash handling procedures do you use for monies received and paid out?	
9. Tell me about your security procedures to make sure drugs and money are secure.	
10. How often do you get inspected by a government or other inspector?	
Miscellaneous	
1. Please describe your role in the drug distribution process.	
2. What are the top two or three factors in having a successful drug distribution process?	
3. What has been your greatest success in distributing medications?	
4. What is your greatest remaining challenge in distributing medications?	
5. Is there anything else about your drug distribution process that I should know about that I have not asked?	

Interviews were recorded on a handheld Zoom H4n digital recorder in .wav format, and converted to mp3 files using the Audacity program. The mp3 files were uploaded to a professional transcription service that transcribed the interviews verbatim and returned them as Microsoft Word files. Analysis was carried out on the Word files.

### Data analysis

Analysis of the transcripts was performed after all data collection was complete. ESA is a multi-step process in which events are first identified, then coded, and finally assembled into a model. The model is then tested for logical consistency. After testing, the final model emerges to describe the system.

In the initial step, the events found in the data were identified and coded. Events were defined as the individual series of elements that are linked together in a temporal context to constitute a sequence [25]. Working independently, each author coded the data to identify specific, individual events that occurred during the drug supply process and created an initial codebook of events. Event codes were derived from the data using a positivist approach [34]. The authors then met to discuss areas of agreement and non-agreement in the events each had coded. The coding schema were adjusted after each discussion. By the seventh iteration, the final codebook emerged and was used to re-code the five transcripts that were judged the most detailed in describing the drug procurement, storage, and distribution process. The remaining transcripts were used to add context and meaning to the events identified in the transcripts used for ESA. The final coding schema represented the authors’ best attempt to be complete, yet parsimonious, with the events identified. The final codes used and a brief description of each are included in Table 3.

**Table 3:** Events used in ESA

Description of Event	Short Name Used in ESA and Model
Establish/create the entire system	Create System
Aboriginal health service chooses community pharmacy and hospital as sources of drug supply	Choose Supplier
Create imprest/formulary system	Create Imprest
Clinics receive budget for non-imprest items	Clinic budget
Aboriginal health service decides on drugs and purchase/order quantity	Order drug & qty
Clinics place order with head office	Clinics Order
Order processed in head office by continuity of care pharmacist	Process in HO
Order processed by community or hospital pharmacy in regional capital city	Process pharm
Processing community or hospital pharmacy purchases drugs from wholesaler	Pharmacy purchase
Wholesaler ships drugs to community or hospital pharmacy	Wholesale Ship
Community or hospital pharmacy fills order and supplies drugs to Aboriginal health service	Pharm Supplies
Community or hospital pharmacy bills PBS under Section 100 or bills Aboriginal health service for non-covered medications	Pharm Bills
Community or hospital pharmacy gets paid by PBS or Aboriginal health service	Pharm Paid
Cold chain protocols employed to package medication for shipping in Eskies and medications shipped outside of temperature range are disposed of.	Cold Chain
Packaged medications brought to airport and transported by air to local clinics.	Air Transport
Emergency or other medications between monthly re-supply flights transported from one clinic to another by RAP	Emergency Process
Medications received in the clinic are unpacked and checked for accuracy.	Clinic checks
Packing slips/invoices sent back to head office for audit purposes. Documents are available for any audit by PBS.	Audit
Rotate stock in clinic. Place new medications behind old medications.	Rotate Stock
Monitor for and dispose of expired medications	Expired Products
New prescriptions written by physician or RAN using CARPA guidelines if no physician available	Rx Written
Medications dispensed to patient by RAN	Dispense Med
Medications packed and dispensed to patients in dosettes if necessary	Pack Dosettes
Patient receives drug from RAN	Drug to Patient
Patient received necessary medication counseling or education	Patient Counsel

ESA was the second step in data analysis, and was used to develop the model. The one or two-word abbreviation (e.g. choose supplier) for each event in Table 3 was entered into ETHNO, which is the computer program used to perform ESA [35]. Events were entered in what the transcripts indicated was the sequential order in which they occurred. The prerequisite analysis form of ETHNO’s linking function was then used to logically connect the events and finally to connect the events into a sequence. The linking function allows the analyst to choose one of four forms of questions ETHNO can ask to link events (prerequisite; implication; historical causation; counterfactual). The analyst can also specify if she wants to begin with the end action or the initial action of the sequence.

In this analysis, the prerequisite form of question was used and commenced with the initial action. By starting with “create system” (the initial action) and asking a pre-requisite form of question, ETHNO looked at the next event entered and asked, “Does ‘choose supplier’ require ‘create system’ or a similar action?” For this question, the authors chose “yes” and ETHNO asked the same question about the next event. Thus, ETHNO asked, “Does ‘create imprest’ require ‘choose supplier’ or a similar action?” The authors chose “no” and ETHNO moved on to the next pairing. ETHO continued this process until all possible linking questions had been asked.

The number of yes/no linking questions ETHNO asks is significantly reduced by the application of syllogistic reasoning. For example, if event Y requires event X and event Z requires event Y, by syllogistic reasoning, event Z requires event X.

After answering the final yes/no question, ETHNO’s chart function was used to create a diagram that illustrates the sequence of how events are related. This diagram constituted a proposed model of the drug distribution system.

ETHNO’s testing function was then used to test the logic of the proposed model. Testing requires the analyst to test the model’s logic against three assumptions programmed into ETHNO. These assumptions are: (1) an event cannot occur until all of its prerequisites are fulfilled; (2) an event depletes the conditions produced by prerequisite events; (3) an event does not repeat unless separated by some consequence of that event.

To illustrate these assumptions, consider for example the events that could occur in the case of a pitcher and a batter. Assumption 1 means the batter cannot hit the ball until the prerequisite of the pitcher throwing it is fulfilled. Assumption 2 means the batter cannot hit the ball again because the pitcher throwing it depletes the event of throwing it. Assumption 3 means the pitcher cannot repeat his pitch until a consequence of the initial pitch, such as the batter hitting the ball back to him, has occurred.

The testing function of ETHNO visually highlights on the diagram instances where any of the above assumptions are not met by the proposed model and suggests possible solutions that allow the analyst to “relax” an assumption. For example, ETHNO may suggest that: (1) one action is not actually a prerequisite for a later action (e.g. the pitcher does not actually need to throw the ball before the batter hits it); (2) an action is not depleted by the prerequisite action (e.g. the batter hitting the ball does not mean the batter cannot hit it again) or; (3) a pre-requisite for the action was not recorded in the analyst’s presumed sequence of events (e.g. the analyst did not record that there is also a catcher who returned the ball to the pitcher after the batter swung and missed). In this example, the first two solutions are logically implausible and the analyst would have to determine if there was a missing event in the sequence being analyzed.

**Fig. 2:**
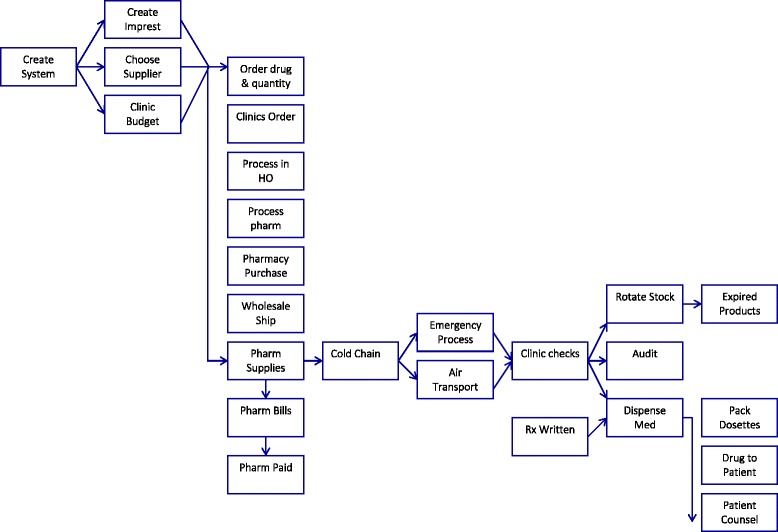
Drug distribution model for remote Australia

After completing the testing function, all problems with ETHNO’s inherent logic were resolved, and the model shown in Fig. 2 emerged.

## Results

Since ESA is a qualitative method, the results of this study can be presented from two different perspectives. The model ETHNO creates provides a visual description of the drug procurement, storage, and distribution system. This model is shown in Fig. 2. In addition, the rich data collected using qualitative methods allows for a deeper analysis that can be used to describe the opinions and perceptions of those who work in the system.

### The model

As shown in Fig. 2, the drug procurement, storage, and distribution system was made up of 24 unique steps. These 24 steps represented seven distinct components described below.

1. *Choosing a supplier -* Since the health service was not a licensed pharmacy, the first step was to choose a medication supplier. The primary source chosen was a community pharmacy 600 miles away. The hospital in a regional capital city also supplied some medications not always found in a community pharmacy.
2. *Creating list of preferred medications -* Creating an “imprest” list meant the health service developed a formulary of preferred drugs that qualified for an additional subsidy under Section 100 (S100) of the Australian Pharmaceutical Benefits Scheme (PBS) [36]. The PBS subsidizes medication costs for all Australians. Under S100, medications are usually free to patients who qualify.
3. *Budgeting and ordering -* Each clinic was given a budget to purchase medications or supplies not on the imprest. Their spending patterns were compared to their patient loads as a monitoring tool. Based on previous usage, clinics were also provided with a recommended order quantity. The RAN reviewed the drugs and quantities in stock monthly, and placed an electronic order with the head office in the regional capital city. The order was reviewed and processed by the continuity of care pharmacist and sent to the supplying pharmacy to be filled.
4. *Supply and shipping -* Depending on its inventory, the supplying pharmacy purchased any necessary medications from its wholesaler, who shipped them to the pharmacy, which billed the PBS under S100, and was paid for the drug costs and related dispensing fees. Any items (e.g. cholecalciferol) not covered under S100 were billed to and paid for by the Aboriginal health service. Patients at the point of care typically did not pay for their medication.
  Given the intensely hot climate of central Australia, the supplying pharmacy used a cold chain protocol to ensure the stability of medications in transit. Medications susceptible to heat were packed in insulated coolers (known locally as “eskies”) along with one or more ice packs. Temperature monitoring devices were also packed to indicate if the medications had been damaged either due to heat or freezing in transit. Protocols were established for clinics to discard medicines damaged by exposure to temperature extremes.
  Eskies were taken to the airport and stored in a cold room overnight. They were loaded onto a small plane contracted by the Aboriginal health service. Since medications could be needed between scheduled re-supply flights, an emergency drug supply process was created. Typically, this meant the RAP drove several hours to pick up a medication from one clinic to deliver it to another.
5. *Receipt and storage in the clinic -* Clinic staff met the plane, off-loaded the medication immediately, and brought it to the air-conditioned clinic. The eskies were unpacked and the clinic staff checked the contents to ensure the correct medications had been delivered in the correct quantities and were undamaged in transit. A copy of the invoice was sent to head office to be audited for completeness and accuracy by the continuity of care pharmacist.
  In the clinic, medications were stored in a locked, secure room. The clinic staff placed newer medication behind current inventory. As medications were dispensed, newer packages slid down the angled shelves to replace older stock. Clinic staff were required to check inventory monthly, follow an expired products policy to rotate stock as necessary, and discard expired products by sending them back to head office by truck for disposal.
6. *Prescribing processes -* Medications were prescribed and supplied by several processes. Chronic medications were dispensed by the RAN as patients required. Usually, new medications were verbally prescribed by a physician, after a telephone consultation with the RAN. Some new medications were prescribed directly by the RAN under guidelines developed by The Central Australian Rural Practitioners Association (CARPA) [37].
7. *Dispensing and patient counseling -* Depending on the patient, clinic staff repackaged the medication into a dosette box to promote adherence. The patient was then given the dosette and provided with medication counseling as necessary.

### Health care worker opinions and experiences for quality assurance and policy making

The opinions and experiences of health care workers who worked in the model would be of interest to the Aboriginal health service’s administrators, as well as policy makers charged with determining how best to ensure all patients had safe and convenient access to medications. Although not a formal quality assurance process, knowing what one’s employees think works or does not work well, and getting constructive criticism, is helpful for system design or benchmarking. Interview results made it apparent that, although the system was well designed and functional, there remained areas for improvement.

#### Choosing a supplier

By outsourcing drug supply, the health service risked experiencing poor service and needing to find another supplier. This proved to be problematic.

“A pharmacy owned by a previous pharmacist had supplied [redacted] for about 20 years, so we just kept going. They gave good service. It was bought out by [redacted], who have an interest in all the pharmacies, so it really doesn’t matter which pharmacy you go to; you get the same.”

Interview #6

From a policy perspective, this information is useful to review pharmacy ownership legislation and its impact on patient care.

#### Legal issues and staffing

In this model, the legal requirements for medication handling were inconsistent with the capacity of the health service to meet them. Having a physician write all new prescriptions was legal, but not feasible. Having the RAN prescribe medications for acute illnesses was feasible, but not legal. The RAN’s primary role was to educate and monitor patients, and resupply their medicines as necessary. In acute cases, a physician was supposed to prescribe a medication after a remote consultation with the RAN, but this was often not possible. The underlying problem appeared to be an insufficient number of physicians available to meet the patient demand.

**“….** we follow the CARPA protocol, which is basically that you’ve got protocols, protocol-based prescribing. They have to do that because we’ve got one doctor every second month. If we look at the disease states and you follow some sort of [Inaudible]…., we need 12 doctors on the Lands. We’ve got one every second month. If we’ve got to have every person with pneumonia to be seen by a doctor, remote health, that was [Inaudible]. Nurses prescribe based on the protocol for that for the time. When it comes to regular chronic meds, then there’s no prescriptive rights; that’s doctor only….. It’s not quasi-legal, but we feel if worse comes to worst we can set up a defensible standard of practice to say why this is followed.”

Interview #6

To a policy maker, these results may reveal the need to create a waiver for prescribing and drug supply policies in remote areas. A waiver would allow physicians, nurses and pharmacists to practice within the law, while providing access to necessary medications.

#### Cold chain breakdowns

The cold chain in this model was complex and prone to breaking down.

“I’ve put ice bricks down in the bottom, as well as the top, so we’re trying… Because the last esky I sent, it actually got hot, so now I’m trying to put some on the bottom as well as on top…. That was really good because that was a trial for me to see if it’s going to freeze or not…… In winter, a lot of times, we get a few frozen, so that’s quite hard to know, do you put two? .... it’s quite hard to know. We do try and make sure it’s packed well enough to, hopefully, keep from freezing or over- heating.”

Interview #8.

“The cold chain, that’s our Achilles’ heel …. One plane a week is not enough to carry stores, mail….. when they have to take passengers, they offload stores and they don’t discriminate what they offload.”

Interview #5.

#### Medication shortages and wastage

The imprest list and tailoring the order quantity to each community was expected to reduce wastage and ensure that patients always had access to their medicines. Yet, this was not always the case. If there was a death in the community, Aboriginal culture required the community to set up a “sorry camp” and the population of the community could increase substantially and result in medication shortages.

“If somebody dies in this community, …. everyone moves out of their houses and lives in the camp in the bush just back here. People will come from other communities, and it’s their way of sharing the grieving, I suppose…... You might have a community like this of 120 people and if someone was to die, by Monday we could have 300. That’s a possibility, especially if it’s an old senior person. That could definitely double your numbers on a weekend.”

Interview #5

Medication wastage remained a problem even with an order quantity that was specific to each clinic.

“There’s a lot of wastage…..the one thing we do often have a problem with is stock may come out that doesn’t have the longest of expiry dates on it….. you can end up sometimes discarding stuff because it has expired before it’s had a chance to be used.”

Interview #4

#### Adherence to policies

Proper functioning of the system required workers to perform all the tasks required by the system. Although the model required the RAN to check for, and dispose of expired medications on a monthly basis, it did not always happen.

“In actual fact, our protocol is we should be checking every month and delete your out-of-date stuff. I don’t know that it happens religiously.”

Interview #1.

Similarly, the audit process was problematic, because the RAN did not always comply with the requirement to send the invoice received with their order back to head office.

“The nurses are supposed to do all the invoicing when it comes in. Sometimes the copy of what’s supplied hasn’t arrived with the order. Most times the nurses don’t do it and don’t send it back to the office…... The nurses just put it on the shelf. My order was out, but they can’t tell me why and they haven’t checked the invoice off.”

Interview #7.

For policy makers within the Aboriginal health service, these results make it clear that adherence to existing policies is inadequate. They must therefore decide to either enforce the current policy more stringently, adopt other policies that the RAN will adhere to, or create new personnel management policies in which there may be consequences for RANs who do not adhere to the required protocols.

## Discussion

Studies such as this may have several applications. They may be useful to evaluate how successfully a pharmacy system addresses access to medication as one of the social determinants of health. In addition, the sparse literature that describes drug distribution systems in underserved areas means that health system managers and policy makers have few resources to assist them. Such individuals have two tasks for which studies such as this may be applicable. They must design a drug procurement, storage, and distribution system, and once the system is in place, they must be able to evaluate it for quality assurance purposes. These results may also be relevant to faculty in health professions education programs. Such programs increasingly offer their students opportunities to serve on “medical mission” trips [38–41]. Students and faculty trip planners will need to determine the best way to obtain and distribute medications to the patients they will serve.

### Social determinants of health

Health systems, in which ready access to medications is a fundamental component, are one example of a social determinant of health [2, 3]. In this model, the drug procurement, storage, and distribution system was found to be comprised of a 24-step process that could be broken down into seven components: choose a supplier; choose a list of preferred medications; budgeting and ordering; supply and shipping; receipt and storage in the clinic; prescribing processes; dispensing and patient counseling. Since these components were able to ensure access to medicines, these results would support an assertion that the system met the requirements set out by Australian accrediting and professional bodies and that pharmacists play an integral role in the social determinants of health [6, 7]. Individuals working in this system had the necessary understanding of the sale and supply of medicines, labeling, supply chain management, medication stability, and rural and remote health systems, including service to Aboriginal peoples. Although interviewees were able to identify several areas for improvement the system functioned well, and patients had access to necessary medications.

The model in Fig. 2 appeared to be a successful attempt to ensure that one social determinant of health, safe and convenient access to medications as defined by WHO and Healthy People 2020, was not a barrier to adequate health care [1, 2]. As pharmacists continue to expand their role in the health care system, additional studies that evaluate how well drug distribution systems address other social determinants of health would be welcome.

### System design

A drug supply systems designer is advised to look at the 24 inter-related steps in this model to determine which events, and in which order, are best suited to the system she is creating. How would eliminating, adding or moving one of the steps affect the proposed system?

Or, the designer could look at each step and consider what options are available and their consequences. Choosing a source of medication supply has two likely options – acting as one’s own supplier or outsourcing it.

In this study, the health service determined that running an in-house pharmacy would have onerous legal, storage, inventory, and other requirements. Any potential convenience of an in-house pharmacy, however, had to be balanced against some disadvantages. They were dissatisfied with the level of service provided by their pharmacy supplier and had limited options since their only alternative was to move their supplier 1200 miles from head office. This would then have the knock-on effect of also needing to coordinate with a new hospital to supply vaccines, and a new shipping procedure with another airline.

Similarly, outsourcing shipping to an airline led to problems with the cold chain, and problems when necessary medications were offloaded, or not packed in time to meet the flight schedule. The advantages and disadvantages of multiple shipping alternatives must be considered. Simply choosing the fastest or cheapest option may not be the best one.

A system designer must also consider the relevant legal requirements. This can become complicated, as the system described in this study had its administrative offices in one Australian territory, while the clinics it operated were in an adjacent state. The prescribing process chosen faced legal constraints the system could not readily address. CARPA guidelines were offered as a justifiable alternative to having too few prescribing physicians. Given what one interviewee described as “the tyranny of distance”, no inspector had visited any of the clinics to determine adherence to legal requirements. The prescribing process designed for the system worked, but at a risk. Legal problems could ensue should an audit occur. Additionally, if nurses were prescribing beyond their scope of practice, patient safety could be compromised.

As health care technology continues to advance, systems planners may wish to evaluate if there are technological solutions (e.g. tele-health, drone delivery directly to patients) to some of the problems described above. Although this study did not explore these kinds of alternatives, it is not immediately clear that technology would solve the system’s problems. The population is quite mobile which makes direct delivery to patients impractical [42–44]. Rural Aboriginal people are often culturally not well connected with more urbanized cultures. They may not have a cultural concept of chronic diseases and compliance with medications is often quite poor. How such patients would react to a more technological delivery of healthcare is an open question.

Although these examples may not necessarily be applicable to other remote pharmacy practices, they illustrate how much thought a systems designer must devote to establishing a well-functioning system. A system that works well on paper is still susceptible to problems created by external factors that may not be predictable. Systems designers are encouraged to think broadly, identify as many steps in the drug supply process as possible, spot potential vulnerabilities, and create alternative processes well in advance of any potential system failure.

### Policy making

The opinions and experiences of the various health care providers who work under the model provide several areas that may be useful for policy review. The providers were dissatisfied with the level of service provided by the community pharmacy that served as the primary source of supply. But since all pharmacies in the city were run by the same ownership group, the health service had very few alternatives to choose from. Policy makers would be advised to re-evaluate pharmacy ownership regulations to increase competition among supplying pharmacies and hopefully improve service.

Similarly, new prescriptions for acute conditions were often prescribed by the RAN because there were too few licensed physicians to meet patient demand. Two policy alternatives may mitigate this problem. Prescriptive authority could be delegated to RANs by means of a waiver for nurses in such remote locations who follow CARPA guidelines. Alternatively, a policy decision could be made to increase spending on Aboriginal health services to hire more physicians so RANs could practice within the law.

### Benchmarking and quality assurance

Although this study did not specifically set out to evaluate the quality of the drug distribution system, ESA allowed for a richer analysis of the experiences and opinions of those working in it. These results are not a substitute for a formal quality assurance effort, such as that advised by WHO for pharmacy systems in underserved areas, but the ESA method used in this study may be useful to plan a quality assurance project [45].

Transcripts indicated that health workers identified several areas that could benefit from formal quality assurance. Interviewees expressed considerable dissatisfaction with the supplying pharmacy. This qualitative information could be used to design a more rigorous or quantitative study into how often the supplying pharmacy failed to provide a requested product, or how often shipments arrived with temperature damage. Similarly, how often did shipping by air result in medication being offloaded? Knowing the RAN did not always return the invoice back to head office meant that quality assurance data related to correct quantities being ordered and shipped were not readily available.

For health administrators working in existing systems, having a clear picture of what a functioning drug procurement, storage, and distribution system looks like allows them to benchmark their systems. Knowing all the steps required to get medication from a city to a remote, medically underserved population will assist for such benchmarking.

These examples demonstrate the need for quality assurance studies for a drug distribution system. They also illustrate that high-quality data for such evaluations are as likely to come from people working in the system as they are from the more usual, quantitative metrics.

### Health professions education

Schools of health professions increasingly offer their students an opportunity to provide health services in a variety of rural and remote settings under the supervision of a team of licensed providers [38–41]. Establishing a process whereby the team procures, stores, and distributes medications to their patients is an important part of trip planning [41]. Frequently, the team purchases their medicines in their home country and transports them to the host country as part of their baggage allowance. The risks of this method include lost luggage, having medications confiscated by customs agents, or being charged substantial import levies by the host country.

Trip leaders planning medication purchasing and distribution processes should also consider the option of learning how medicines are purchased and distributed in the host country and partnering with an organization in the host country for medication purchases. Buying medicines in the host country supports the local economy and avoids some of the risks of bringing large amount of medicine from home, but is only an attractive option if medicines in the host country are of good quality [46]. Understanding the system in the host country and the risks and benefits of various ways of purchasing, storing, and distributing medications will increase the chances of a successful service trip.

## Limitations

This study may have several limitations. Qualitative methods are susceptible to social desirability bias by interviewees. This seems unlikely in this study since data saturation occurred early in the data collection process. All interviewees provided broadly similarly responses.

The data reflect the system and experience of one system at one point in time. Policies, procedures and legislation all affect the drug distribution process, and may have changed since the data were collected.

Finally, the model described has not been validated elsewhere by experts in drug distribution systems in underserved and remote areas. Additional research may be required to update and validate the proposed model.

## Conclusions

Drug distribution systems in rural and remote areas are complex, and there is relatively little information available to guide health system designers to create or evaluate systems. The system described here is able to address effectively one of the social determinants of health – adequate access to medications. This study offers evidence that the drug procurement, storage, and distribution system was successfully designed to ensure that people who live under difficult socio-economic conditions, in a rural and remote setting, still have access to necessary medications.

This study illustrates one potential system that can be used to design or benchmark other models. ESA was found to be a useful method to describe a drug distribution model, while rich, qualitative data may be helpful for quality assurance studies after a model has been designed and implemented.

## Abbreviations

CARPA: Central Australian Rural Practitioners Association
ESA: Event Structure Analysis
FIP: International Pharmacists Federation
NGO: Non-governmental organization
PBS: Pharmaceutical Benefits Scheme
PSA: Pharmaceutical Society of Australia
QUM: Quality Use of Medicines
RAN: Remote Area Nurse
RAP: Remote Area Pharmacist
RFDS: Royal Flying Doctor Service
S100: Section 100
WHO: World Health Organization
